# Giant enhancement of tunable asymmetric transmission for circularly polarized waves in a double-layer graphene chiral metasurface

**DOI:** 10.1039/c9ra05760a

**Published:** 2019-10-21

**Authors:** Jiaxin Zhou, Yueke Wang, Mengjia Lu, Jian Ding, Lei Zhou

**Affiliations:** Optical Information Science and Technology Department, Jiangnan University Wuxi Jiangsu 214122 China ykwang@jiangnan.edu.cn; Optoelectronic Engineering and Technology Research Center, Jiangnan University Wuxi Jiangsu 214122 China

## Abstract

In this letter, we propose a structure based on double-layer graphene-based planar chiral metasurface with a J-shaped pattern to generate asymmetric transmission for circularly polarized waves in the mid-infrared region. Asymmetric transmission of the double-layer structure can reach to 16.64%, which is much larger than that of the monolayer. The mechanism of asymmetric transmission is attributed to enantiomerically sensitive graphene's surface plasmons. Besides, asymmetric transmission can be dynamically tuned by changing the Fermi energy and is affected by intrinsic relaxation time. All simulations are conducted by the finite element method. Our findings provide a feasibility of realizing photonic devices in tunable polarization-dependent operation, such as asymmetric wave splitters and circulators.

## Abstract 2

In this paper, we propose a structure based on double-layer graphene-based planar chiral metasurface with J-shaped pattern to generate asymmetric transmission, which can reach to 16.64%.

## Graphical abstract

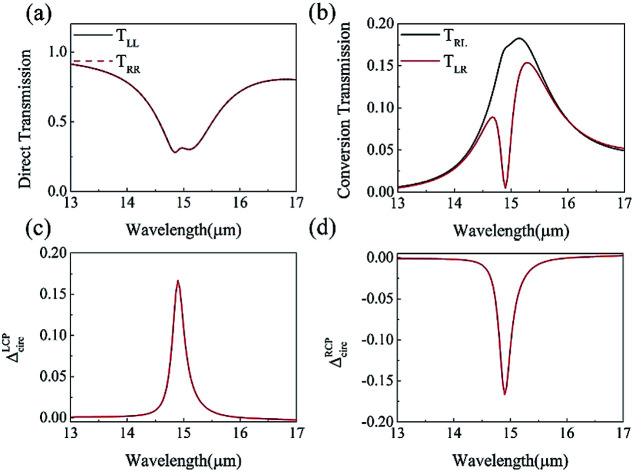

## Introduction

In recent years, chiral metasurfaces have become more and more popular in many fields such as optics. Chirality means that an object cannot be coincident with its mirror image. Existing studies of chiral metamaterials mainly focus on circular^1–4^ and elliptical dichroism,^5^ asymmetric transmission for linearly polarized waves in three-dimensionally chiral planar structures^6–9^ and for circularly polarized waves in two-dimensionally planar chiral structures.^10,11^ Compared with conventional gyrotropy and optical Faraday effects, asymmetric transmission has completely different properties in planar chiral structures. Asymmetric transmission of chiral metasurfaces for circularly polarized waves can be defined as the total transmission difference between the same handedness waves propagating in the opposite directions or the opposite handedness waves propagating in the same direction.^12^ With the development of research on chirality with asymmetric transmission, electromagnetic devices applied for both linearly and circularly polarized waves have been designed, such as some polarization transformers, polarization analysers and polarization-controlled devices.^13–16^ They are widely used to promote the evolution of optical communication and photonics. The excitation of enantiomerically sensitive surface plasmons is a collective mode of electron oscillation at the interface of conductor and medium, which is in charge of asymmetric transmission for circularly polarized waves in planar chiral metasurfaces.^17–19^

As a kind of promising electrically tunable plasmonic material, periodical graphene metasurfaces can excite graphene surface plasmons in the mid-infrared and terahertz (THZ) region.^20–22^ And for graphene metasurface, novel optical properties are dynamically tunable by changing the Fermi energy through voltage control or the changing intrinsic relaxation time through chemical doping.^23–26^ Compared with metallic surface plasmons, graphene surface plasmons have the advantages of low loss, and high localization.^27^ Thus, graphene will revolutionize metamaterials and metadevices, and promote the development of nano-optics.^28^ There are plenty of researches on metallic chiral metamaterials,^29^ but they are generally not tunable, which can be easily realized in graphene metasurface. Recently, people propose kinds of monolayer graphene chiral metasurface,^30,31^ and study the asymmetric transmission for circularly polarized waves. The researches of graphene planar chiral structure will deepen the understanding of the interaction between light and matter in planar chiral structure, which can be applied to tunable polarization sensitive devices, and circular polarizers. But the asymmetric transmission of monolayer graphene chiral metasurface is only up to 5%.

In this letter, we propose a double-layer graphene-based planar chiral metasurface structure to achieve giant enhancement of asymmetric transmission for circularly polarized waves incidence in mid-infrared region. In the monolayer metasurface which is composed of J-shaped hollow graphene patterns, the asymmetric transmission Δ*T* can reach a small value of 2.05%. When another J-shaped pattern, which is designed to be rotated by 90 degrees after being mirror symmetric operation with respect to the previous layer, is added, the asymmetric transmission Δ*T* can achieve 16.64%. The mechanism of asymmetric transmission can be explained with the enantiomerically sensitive graphene surface plasmons, which induce the charge-field excitations. Also, the peaks of asymmetric transmission blue shift with increasing Fermi level, and the values of peaks increase with increasing electron scattering time. Finite Element Method (FEM) simulations are conducted to verify our findings.

## Methods and results

**Fig. 1:**
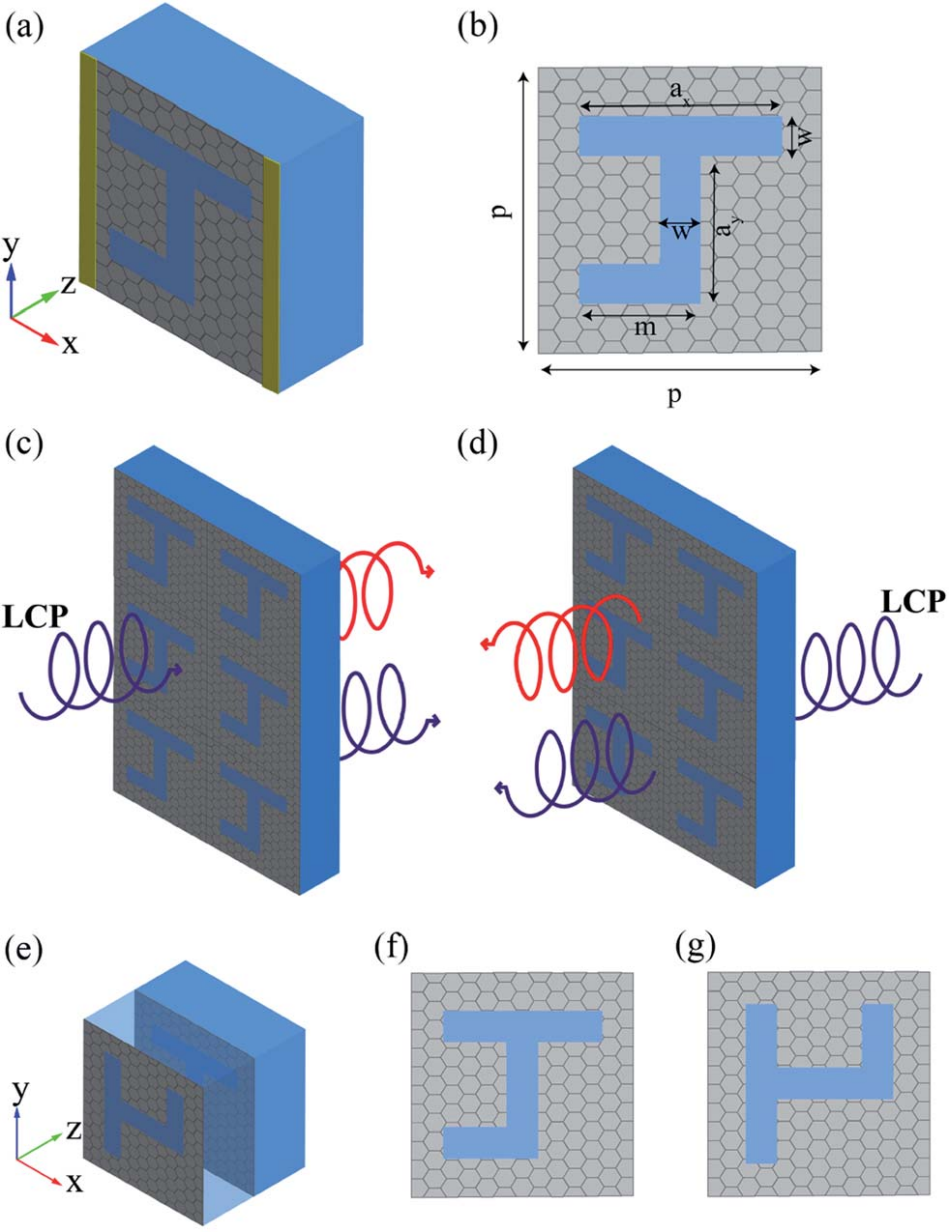
(a) The schematic diagram of one unit of the monolayer graphene-based planar chiral metasurface. (b) The geometric parameters of the hollow J-shaped pattern. LCP incident from the (c) front side and (d) back side of the monolayer graphene-based planar chiral metasurface. (e) One cell of double-layer J-shaped graphene-based planar chiral metasurface. The pattern of (f) the bottom layer and (g) the upper layer.

Firstly, we discuss the monolayer graphene metasurface, and one unit is shown in Fig. 1(a). Here, the graphene sheet is transferred to the quartz substrate, and is decorated with hollow periodic J-shaped patterns. Our proposed structure is two-dimensionally chiral due to non-mirror symmetry, which may lead to asymmetric transmission for circularly polarized waves. Here, the permittivity of the quartz substrate is 2.25. The period *p* of the structure is set to be 270 nm, and the other parameters of the pattern are displayed in the Fig. 1(b). The widths of the top side and the bottom side of J-shaped patterns are *a*_*x*_ = 230 nm and *m* = 125 nm, respectively. The length of J-shaped pattern is *a*_*y*_ = 210 nm, and the width of hollow area is *w* = 20 nm. Periodic boundary conditions are set along *x* and *y* directions for our periodical structure and perfectly matching layers are set in *z* direction to achieve the minimal reflection for the outgoing electromagnetic wave.

According to the Kubo formula, the conductivity of graphene consists of two parts: intraband and interband elements:^32^1
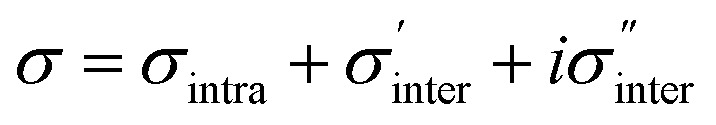
Here, *σ*_intra_ represents intraband conductivity, it can be calculated as:2
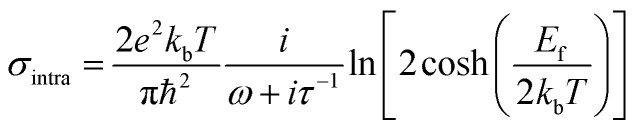
*σ*_inter_ represents interband conductivity, it is characterized by: 
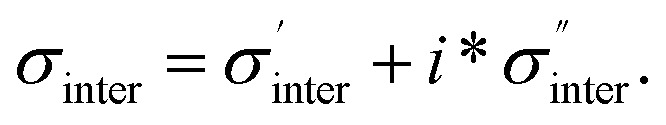
 Here, 
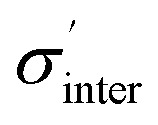
 can be expressed as:3
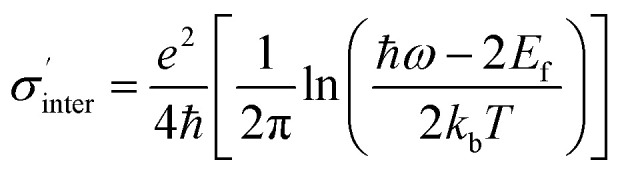



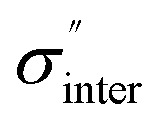
 can be expressed as:4
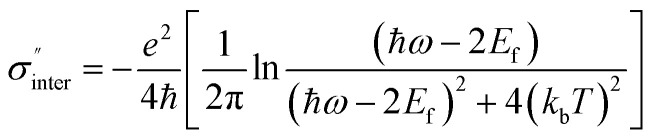
*e* is the electron charge, *k*_b_ is Boltzmann constant, *T* (= 300 K) is temperature, ℏ is Planck constant, *ω* is the angular frequency, and *E*_f_ is Fermi energy. Here, the Fermi energy of the graphene is fixed to be 0.80 eV, and it can be changed by altering the gate voltage applied on the graphene planar metasurface. The relaxation time *τ* is defined as *μE*_f_/*ev*_F_^2^. Here, the Fermi velocity and DC mobility are *v*_F_ ≈ 10^6^ m s^−1^ and *μ* = 10 000 cm^2^ V–^1^ S^−1^, respectively,^22,33^ thus *τ* is 0.8 ps.

The pattern of the J-shaped graphene metasurface has a character of chirality, which can reveal asymmetric transmission when the incident circularly polarized waves have different handedness or propagation directions. Fig. 1(c) and (d) demonstrate the different incident directions of LCP waves, which are coming perpendicularly from the front and the back of monolayer graphene-based planar chiral metasurface. In the complex circular transmission matrices *E*_*i*_ = *t*_*ij*_*E*^0^_*j*_, the indices *i*, *j* represents right-handed circularly polarized (RCP) or left-handed circularly polarized (LCP) components. The transmission matrix elements are defined as *T*_*ij*_ = |*t*_*ij*_|^2^. The circular transmission *T* can be applied to any dispersive optical system under the condition of coherent light incidence. The transmission matrix elements are the same for circularly polarized waves of opposite handedness propagating in opposite directions. It means that the LCP(RCP) waves incident forward equal to the RCP(LCP) waves incident backward. Thus, we can define the transmittances of the LCP waves with opposite propagation directions as

5 *T*^f^ = |*t*_LL_|^2^ + |*t*_RL_|^2^

6 *T*^b^ = |*t*_RR_|^2^ + |*t*_LR_|^2^

*T*^f^ and *T*^b^ represent the transmittances in the forward and backward incident directions for LCP. *T*_LL_ and *T*_RR_ are the direct transmission, and *T*_RL_ and *T*_LR_ are the circular polarization conversion transmission. For our chiral structure with non-C4 symmetry, the direct transmissions of planar chiral metasurface are insensitive to polarization or propagation directions (*T*_LL_ = *T*_RR_). The asymmetric transmission Δ*T* is defined as the difference between *T*^f^ and *T*^b^.

7 Δ*T* = Δ^LCP^_circ_ = *T*^f^ − *T*^b^ = −Δ^RCP^_circ_

Here, Δ^LCP^_circ_ is the asymmetric transmission for LCP incidence, and Δ^RCP^_circ_ is the asymmetric transmission for RCP incidence.

Thus, the asymmetric transmission is only decided by circular polarization conversion transmission.

**Fig. 2:**
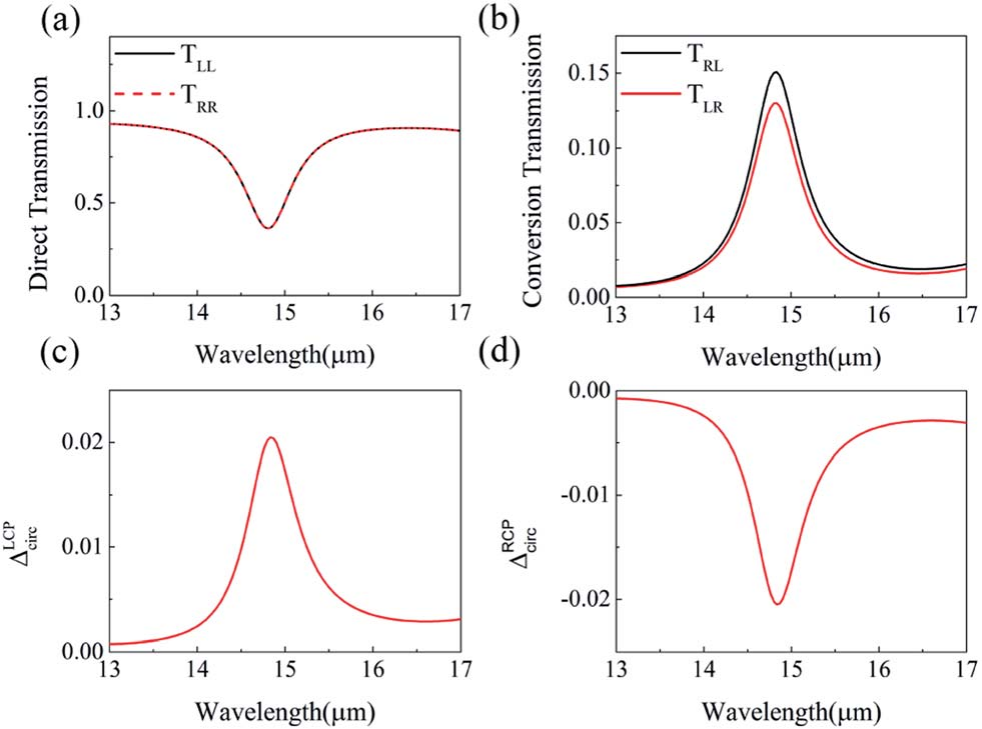
(a) Direct transmission *T*_LL_ and *T*_RR_ and (b) circular polarization conversion transmission *T*_RL_ and *T*_LR_ with wavelength for the monolayer J-shaped graphene metasurface. Asymmetric transmission for (c) LCP waves and (d) RCP waves. The Fermi energy is fixed at 0.80 eV.

Fig. 2(a) and (b) show the calculated results of the four transmission matrix elements when the Fermi energy *E*_f_ is fixed to be 0.80 eV, for the monolayer J-shaped graphene metasurface. Black and red lines represent LCP incidence along the forward and backward directions, respectively. It is found that the direct transmissions (*T*_LL_ and *T*_RR_) always keeps the same with each other with the wavelength changing, and reaches minimum when the incident wavelength is 14.83 μm, shown as Fig. 2(a). It proves that graphene-based planar chiral metasurface is lack of circular dichroism. In Fig. 2(b), *T*_RL_ is always a little larger than *T*_LR_ with the wavelength ranging from 13 to 17 μm, and the maxima of *T*_RL_ and *T*_LR_ are 15.07% and 13.02% at 14.83 μm, respectively. Thus, the asymmetric transmission for LCP waves reaches the maximum value of 2.05% at the wavelength of 14.83 μm, as shown in Fig. 2(c). It is believed that the graphene surface plasmon is resonantly excited along the edges of the hollow J-shaped pattern at 14.83 μm, and in charge of the asymmetric transmission. For RCP incident waves, the asymmetric transmission is quite the contrary to that of LCP incidence based on eqn (7), shown as Fig. 2(d).

Although the asymmetric transmission has been obtained, the Δ*T* is small, compared with that of metallic planar chiral metamaterial structure.^34,35^ To realize larger asymmetric transmission, a double-layer structure is proposed based on the monolayer graphene-based J-shaped metasurface structure. Fig. 1(e) shows the structure composed of two graphene-based planar metasurface layers with J-shaped patterns. The pattern of the bottom layer is shown as Fig. 1(f), which is the same with the monolayer structure of Fig. 1(b); while the upper layer shown as Fig. 1(g) is the pattern of the bottom J-shaped pattern being clockwise rotated by 90 degrees after mirror symmetric transformation about *y*-axis. The medium between the two graphene layers is quartz, and the thickness of the quartz is 125 nm. The LCP waves are still coming perpendicularly from the front and the back of graphene-based planar chiral metasurfaces, respectively.

**Fig. 3:**
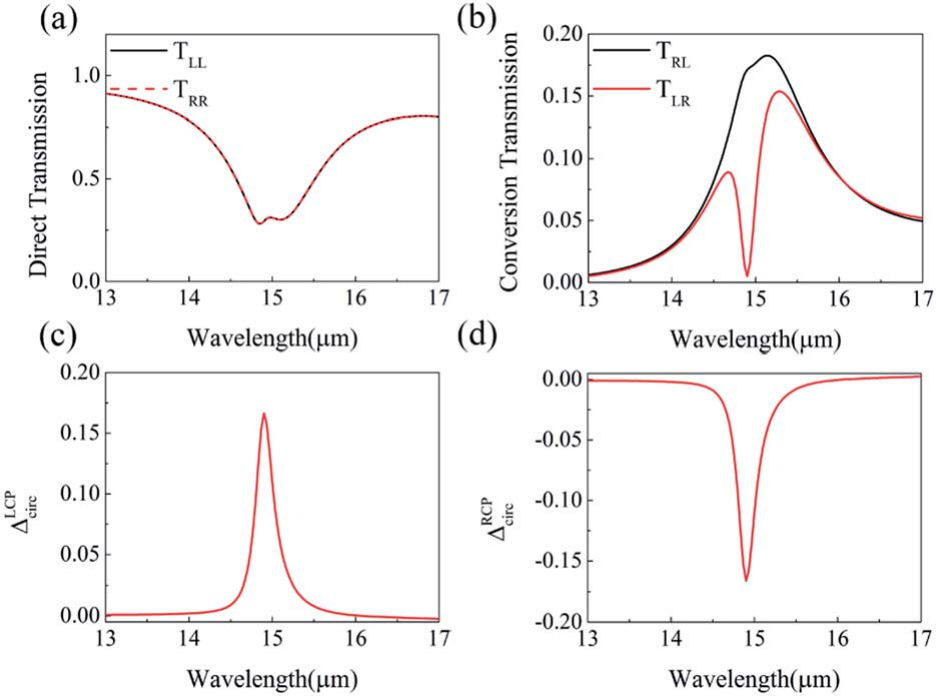
(a) Direct transmission, (b) conversion transmission matrix elements, and the asymmetric transmission for (c) LCP waves and (d) RCP waves of double-layer graphene-based planar chiral metasurface with wavelength changing. The Fermi energy is fixed at 0.80 eV.

Fig. 3(a) and (b) show the calculated results of the four transmission matrix elements, when the Fermi energy *E*_f_ is fixed to be 0.80 eV for the double-layer structure shown as Fig. 1(e). Fig. 3(a) shows that *T*_LL_ and *T*_RR_ are still identical, which proves that asymmetric transmission is independent of direct transmission. In Fig. 3(b), the transmission matrix element *T*_RL_ is slightly higher than that of the monolayer chiral metasurface shown in Fig. 2(b), while *T*_LR_ goes down sharply to almost zero at the wavelength of 14.90 μm. Compared with the monolayer J-shaped metasurface, the resonant wavelength of graphene surface plasmon redshifts a little. Due to the existence of the near to zero value of *T*_LR_, the asymmetric transmission Δ*T* can be increased a lot. As shown in the Fig. 3(c), the maximum of Δ*T* can reach 16.64% at the wavelength of 14.90 μm, which is much larger than 2.05% of the monolayer graphene-based planar chiral metasurface. Similarly, the asymmetric transmission peak of RCP incident waves reaches −16.64%, just the opposite of LCP. The giant enhancement of circular asymmetric transmission suggests that the structure of double-layer J-shaped graphene metasurface can greatly improve chirality.

**Fig. 4:**
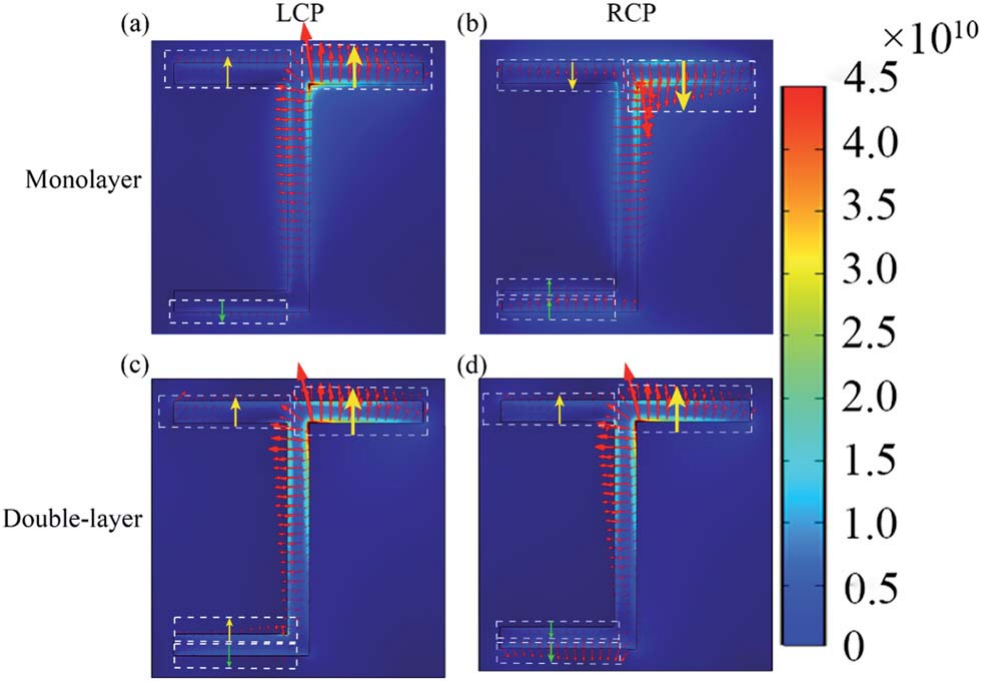
The induced electric field in the hollow area of the bottom layer of graphene chiral planar metasurface at the resonance wavelengths (a) LCP and (b) RCP waves propagating along the forward direction for the monolayer structure when the wavelength is 14.83 μm; (c) LCP and (d) RCP waves propagating along the forward direction for the double-layer structure when the wavelength is 14.90 μm.

In order to understand the physical mechanisms behind the asymmetric transmission, the induced electric field in the hollow area of the bottom layer of graphene metasurface is studied in Fig. 4 and the induced electric vector field is indicated by red arrows. At the resonance wavelength, a standing wave of current oscillations is excited by electromagnetic radiation. This coupled oscillation of the electron density and electromagnetic field is a localized plasmon. Plasmonic excitation of chiral structure is different from that of ordinary metamaterials since it is enantiomerically sensitive.^36^ The excitation level is decided by the direction and polarization state of the incident waves. Here, graphene localized surface plasmon is excited along the J-shaped edge because the electric field is enhanced around the J-shaped edge shown in Fig. 4. Here, the asymmetric transmission is attributed to the enantiomerically sensitive graphene surface plasmons. When the LCP (or RCP) at the resonance wavelength is incident on the graphene metasurface from the forward (or the backward) direction, the enantiomerically graphene surface plasmon is excited, enhances the local electric field, and excites the induced electric field in the substrate, which determines the radiation (direct transmission, and conversion transmission). The striking difference between plasmonic excitations of opposite handedness leads to the different induced electric field. With greater chirality, the induced electric field is more different and the difference of the circular polarization conversion between the opposite handedness will be stronger. The components *E*_*y*_ of the induced electric field for LCP(RCP) incidences can reflect the excitation level of localized plasmon, and explain the difference between *T*_LR_ and *T*_RL_. Here, the yellow and green arrows was painted to demonstrate the components *E*_*y*_ along *y*-directions as shown in Fig. 4. Fig. 4(a) and (b) show the induced electric field distribution under the monolayer graphene metasurface in the substrate. The green arrows for the RCP incidence are stronger than that of the LCP incidence for the monolayer metasurface structure, which indicates the suppression of circular polarization conversion is stronger. Thus, *T*_LR_ is smaller than *T*_RL_, and an asymmetric transmission (Δ*T* = *T*_RL_ − *T*_LR_) of 2.05% is achieved for the monolayer metasurface structure. Fig. 4(c) and (d) show the induced electric field distribution under the double-layer graphene metasurface in the substrate when the incident wavelength is 14.90 μm. The induced electric field components *E*_*y*_ for the LCP incidence indicated by yellow arrows in Fig. 4(c) are stronger than those in Fig. 4(a), which indicates that the suppression of circular polarization conversion is smaller. Thus, *T*_RL_ of double-layer increases to 17.13%, compared with 15.07% of the monolayer. As shown in the Fig. 4(d), more electric fields indicated by the green arrows and less electric fields indicated by the yellow arrows than those of Fig. 4(b) means that the electric field is weakly coupling to free space, scattering is low and suppression of circular polarization conversion is much stronger than that of monolayer metasurface.^17,36^ Thus, *T*_LR_ is near to zero, and the asymmetric transmission of the double-layer metasurface can be greatly increased and Δ*T* can reach 16.64%.

**Fig. 5:**
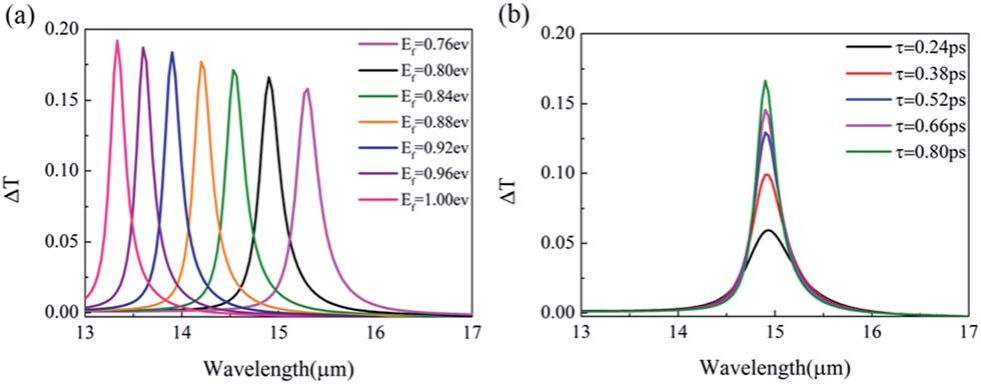
(a) The relation between the asymmetric transmission Δ*T* and wavelength under different Fermi energy. (b) The relation between Δ*T* and wavelength under different scattering time *τ*, when *E*_f_ is 0.8 eV. Other parameters are the same with that of Fig. 1(e).

To study the dynamical tunability of asymmetric transmission, we changed the Fermi energy and intrinsic relaxation time of graphene layers. When increasing the Fermi energy from 0.76 to 1.00 eV, the peak of asymmetric transmission has a blue shift changing from 15.30 to 13.30 μm, as shown in Fig. 5(a). It suggests that graphene-based planar chiral metasurface layer structure has a feasibility of realizing polarization sensitive devices in a wide wavelength range. This blue shift behaviour can be interpreted by the following: the excitation of graphene surface plasmonic wave vector is satisfied with the equation *K*_spp_ = *ħω*^2^/2*α*_0_*E*_f_*c* ∝ 1/*L*_g_^37^ where *α*_0_ = *e*^2^/*ħc* is the fine structure constant. The resonant wavelength λ can be written as:8
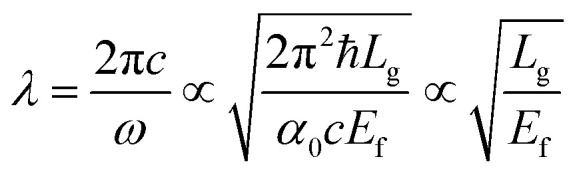
Here, *L*_g_ represents the resonant characteristic length of the graphene hollow pattern. Consequently, it is clearly demonstrated that increasing Fermi energy *E*_f_ can cause blue shift of these resonant wavelengths. In addition, when Fermi energy increases, the peak amplitude of asymmetric transmission increases from 15.82% to 19.19%. Fig. 5(b) shows the relationship between intrinsic relaxation time *τ* and asymmetric transmission, when the *E*_f_ is 0.80 eV. Here, *μ* is usually in the range of 3000 cm^2^ V^–1^ S^−1^ to 10 000 cm^2^ V^–1^ S^−1^, so intrinsic relaxation time *τ* can vary from 0.24 to 0.80 ps. With the increase of intrinsic relaxation time *τ*, the peak value of the asymmetric transmission increases from 5.95% to 16.64%, and the peak position remains unchanged, always at the wavelength of 14.90 μm. This increase of the asymmetric transmission peak value with increasing electron scattering time *τ* is caused by the reduced free-carrier loss in graphene, which can lead to a increase in the circular polarization conversion transmission *T*_RL_ but no change of *T*_LR_. Therefore, the frequency peak position of asymmetric transmission can be tuned because the Fermi energy can be changed by tuning the gate voltage, compared with the metallic chiral metamaterials. For tuning the Fermi energy of graphene in the potential experiments, we can cover the structure with a layer of ion gel following the procedure described in ref. 38. A top gate gold contact is fabricated on the ion gel layer and the bottom gate is connected to silica bottom. The fermi energy of the graphene nanostructure could be tuned by applying a bias voltage.^39^

**Fig. 6:**
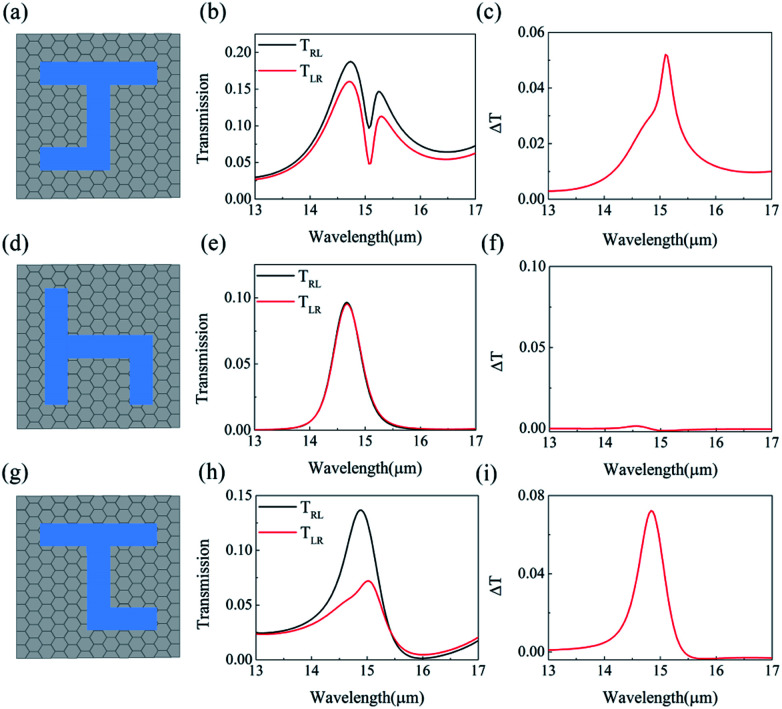
The upper patterns of the three typical kinds of double-layer structures, which are the same with the bottom layer pattern (a), the bottom layer pattern being rotated by 90 degree (d) and the bottom layer pattern being mirror rotated about *y*-axis (g), respectively. The conversion transmissions (b), (e) and (h) and the asymmetric transmissions (c), (f) and (i) for structures of (a), (d), and (g), respectively.

Other double-layer structures of J-shaped pattern have been also studied, and circular polarization conversion are calculated, when *E*_f_ is fixed to be 0.80 eV. Fig. 6(a), (d) and (g) show the upper patterns of the three typical kinds of double-layer structures, which are the same with the bottom layer pattern, the bottom layer pattern being rotated by 90 degrees and the bottom layer pattern being mirror rotated about *y*-axis, respectively. Fig. 6(b), (e) and (h) show the circular polarization conversion of the three kinds of two-layer structures, it is found that the difference between *T*_LR_ and *T*_RL_ varies with wavelength. For the structures of Fig. 6(a) and (g), the asymmetric transmission can reach maximum 5.19% and 7%, when *λ* locates at 15.1 and 14.8 μm, shown as Fig. 6(c) and (i). And for the structure of 90 degrees rotation, the asymmetric transmission almost disappears. Thus, the largest asymmetric transmission can be obtained for the double-layer structure of Fig. 1(e).

## Conclusions

In summary, we demonstrated and studied the double-layer graphene-based planar chiral metasurfaces structure. Through FEM numerical simulation, we chose the most suitable orientation of double-layer graphene pattern, and the asymmetric transmission can reach to 16.64%, which is much larger than that of the monolayer. In addition, the induced electric field below the graphene chiral metasurfaces are analyzed for understanding the physical mechanism of asymmetric transmission. We attribute the asymmetric transmission for circularly polarized waves to the enantiomerically sensitive plasmonic excitations. Besides, the peak of asymmetric transmission could have frequency shift and amplitude changes when the Fermi energy and intrinsic relaxation times of graphene are changed. We believe our findings can be applied to tunable polarization sensitive devices, circular polarizers and other fields.

## Conflicts of interest

There are no conflicts to declare.

## Supplementary Material
